# Underlying mechanisms of acupuncture therapy on polycystic ovary syndrome: Evidences from animal and clinical studies

**DOI:** 10.3389/fendo.2022.1035929

**Published:** 2022-10-24

**Authors:** Yang Ye, Cong-Cong Zhou, Hang-Qi Hu, Ii Fukuzawa, Hao-Lin Zhang

**Affiliations:** ^1^ Department of Traditional Chinese Medicine, Peking University Third Hospital, Beijing, China; ^2^ School of Global Public Health, New York University, New York, NY, United States

**Keywords:** acupuncture, animal studies, clinical studies, mechanism, polycystic ovary syndrome, review

## Abstract

Polycystic ovary syndrome (PCOS) is a common endocrine and metabolic disorder among women of reproductive age. Current standard treatment includes lifestyle change, oral pharmacological agents, and surgical modalities. However, the efficacy of current therapies is less than satisfactory. Clinical evidence has shown that acupuncture is effective for regulating hormone levels, promoting ovulation, and attenuating insulin resistance in patients with PCOS. Acupuncture may affect the production of β‐endorphin, which may lead to gonadotropin-releasing hormone secretion and then affect ovulation, menstrual cycle, and fertility. The mechanism of acupuncture for patients with PCOS has not been comprehensively reviewed so far. Better understanding of the mechanisms of acupuncture would help popularize the use of acupuncture therapy for patients with PCOS. In this narrative review, we aimed to overview the potential mechanisms and evidence-based data of acupuncture on PCOS, and analyze the most frequently used acupoints based on animal and clinical studies. The results of this study will contribute to a better understanding of the current situation in this field.

## Introduction

Polycystic ovary syndrome (PCOS) is a common endocrine-metabolic disorder in reproductive-aged women, affecting up to 15% of reproductive age women (1), and has become the leading cause of menstrual disorders and anovulatory infertility in women (2, 3). The major clinical manifestations of PCOS consist of ovulatory dysfunction, hyperandrogenism, and polycystic ovaries, along with insulin resistance, obesity, and metabolic dysfunction (4, 5). In addition, PCOS is also linked with other complications, such as type 2 diabetes, endometrial dysfunction and cancer, cardiovascular disease, depression, and pregnancy complications (6–10). The current standard treatment for PCOS includes lifestyle change, pharmacological therapy, and surgical modalities, but the effect is less than satisfactory (11, 12). Lifestyle change is the first-line therapy for PCOS patients, especially for overweight and obese women. However, this is a very difficult task for many people (13). The main pharmacological therapy for PCOS patients is the oral selective estrogen receptor modulator like clomiphene citrate (CC). CC is ineffective in 40% of PCOS patients and is associated with significant side effects, such as headaches, bloating, mood swings, and breast tenderness (14, 15). Letrozole is considered the first-line treatment to induce ovulation, with the ability to improve clinical pregnancy rates and reduce time-to-pregnancy in women with PCOS (15). However, it is associated with higher risks of hot flashes, arthralgias, fatigue, and myalgias (16). Metformin, an insulin sensitizer, has been widely used for the treatment of PCOS patients (17). It increases insulin sensitivity, but associated with unsatisfactory weight loss and increased risk of hypoglycemia (18, 19). Gonadotropin has been proven to be effective in ovulation induction. But it may lead to the development of multiple follicles and increase the risk of ovarian hyperstimulation syndrome (20). Therefore, there is a need for a new therapy, which should be inexpensive, easily administered, and free of serious side effects.

Acupuncture has been used as a medical means in China for thousands of years (21). The use of acupuncture in the reproductive endocrinology and infertility of PCOS has recently gained increased popularity worldwide. Several clinical trials have shown that acupuncture may have beneficial effects on ovulatory dysfunction and infertility in patients with PCOS. Acupuncture has also been reported to improve insulin sensitivity and decrease testosterone in patients and animals with PCOS (22). However, there is an insufficient amount of research evidence to support the clinical efficacy of acupuncture treatment for PCOS women, and the mechanisms of acupuncture are unclear, previous clinical and experimental studies indicate that acupuncture influences PCOS-like symptoms *via* various mechanisms. Recent reviews have demonstrated that acupuncture adjusts hormone levels by regulating hypothalamic-pituitary-ovarian (HPO) axis or regulating levels of anti-Müllerian hormone (AMH) and P450arom. A recent systematic review on effect of acupuncture on PCOS in animal models summarized that acupuncture could improve insulin resistance by upregulating the insulin receptor substrate-1 (IRS-1)/PI3K/glucose transporter 4 (GLUT4) pathway, or inhibiting the PI3K/AKT/mTOR pathway, or activating the adenosine 5’-monophosphate activated protein kinase (AMPK) pathway in PCOS animals (23).

Until now, no review has been published to summarize the effects of acupuncture both in PCOS patients and animal models. Therefore, the present study aims to review the potential mechanism and evidence-based data of acupuncture on PCOS, and analyze the most commonly used acupoints. The results of this narrative review may provide directions for future research in this area.

## Searching methods

### Search strategy

A comprehensive literature search was performed by two independent investigators in PubMed, Web of Science, and Scopus databases from establishment to July 2022 to identify related publications. The advanced search option was used to select relevant keywords and identify Medical Subject Headings (MeSH) terms [i.e., (Acupuncture* OR Electroacupuncture*) AND (Polycystic Ovary Syndrome* OR Polycystic Ovarian Syndrome* OR PCOS*)].

### Eligibility criteria

All human and animal studies concerning the effect of acupuncture on PCOS treatment were included in this review. There was no country restriction, but the language was limited to English. Moreover, no restrictions were imposed involving publishing date, type of subjects or type of reported outcomes. Researches were excluded if the type of intervention was moxibustion or transcutaneous electrical acupoint stimulation or acupressure.

### Study selection

**Figure 1:**
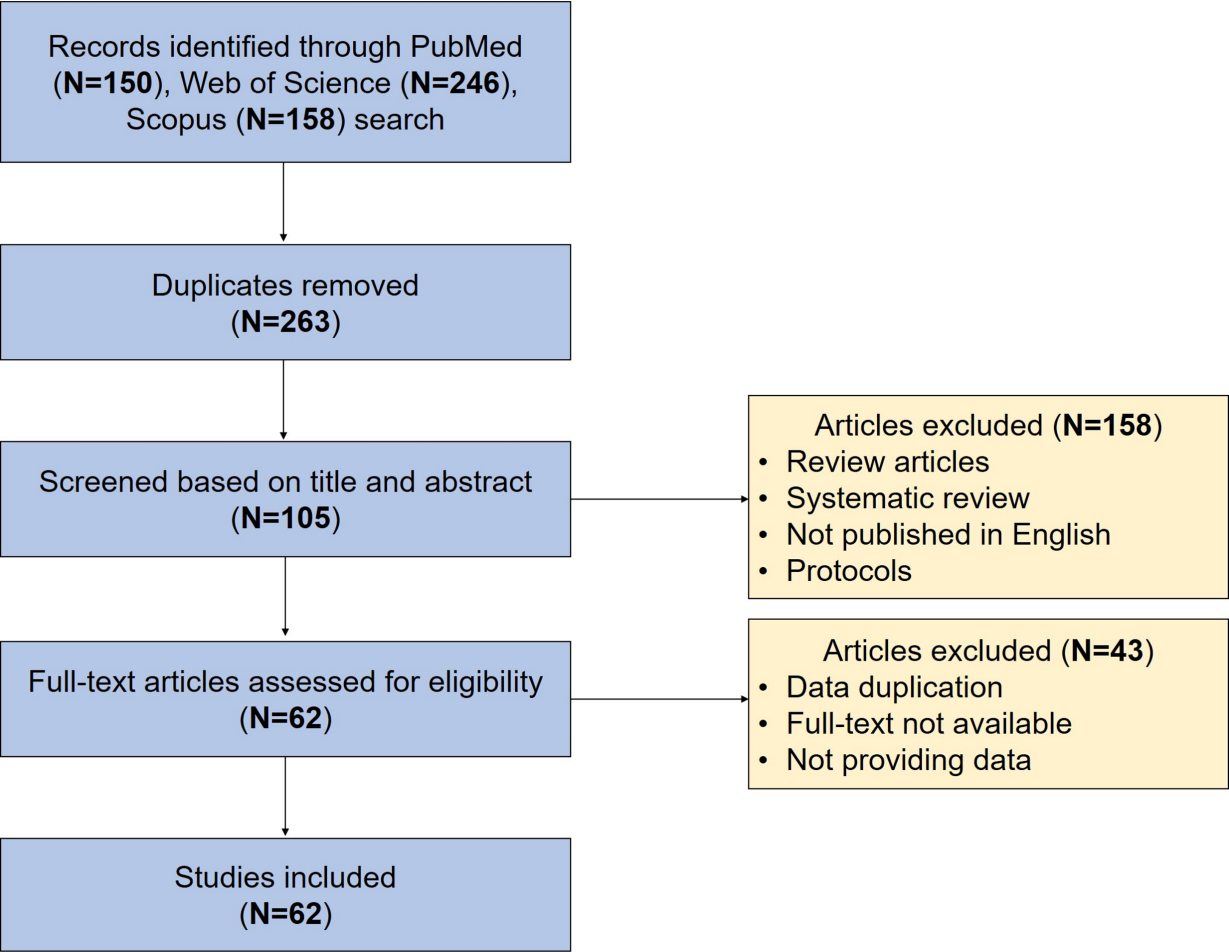
Flow chart of the study selection process.

The following assignments were independently accomplished by two investigators. We identified 150 articles on PubMed, 246 articles on Web of Science, and 158 articles on Scopus. After duplicates were removed, 263 articles conformed to our search criteria. The titles and abstracts of the identified articles were reviewed, and irrelevant search results were deleted. After that step, the full text of the remaining literature was assessed. A total of 62 publications were finally included in this review (**Table 1**). Among them, 28 publications were animal studies, and 34 publications were clinical studies. The flow chart of the screening process is shown in **Figure 1**.

**Table 1:** Characteristics of acupuncture in the treatment of PCOS from main studies included.

Year	Author	Subject	Intervention type	Acupoints	Frequency and Duration	Mechanisms
2000	Stener‐Victorin (24)	PCOS rats	EA	NR	every second or third day up to 12 times	↓ovarian NGF concentrations
2003	Stener‐Victorin (25)	PCOS rats	EA	NR	12times	↓ NGF, endothelin 1
2004	Bai (26)	PCOS rats	EA	SP6, E128	2 per wk for 8 wks	reversed the NGF abundance
2005	Manni (27)	PCOS rats	EA	NR	every second day	↓beta2-ARs mRNA
2008	Manneras (28)	PCOS rats	EA	ST29, SP6	every second weekday for 4 –5 wks	↑insulin sensitivity
2009	Feng (29)	PCOS rats	EA	ST29, SP6	5 per wk for 4-5 wks	↓hypothalamic GnRH and AR expression levels
2009	Manneras (30)	PCOS rats	EA	NR	every second weekday for 4–5 wks	↓expression of genes encoding markers of sympathetic activity
2009	Stener‐Victorin (31)	PCOS women	EA	CV3, CV6, ST29, SP6, SP9, LI4, PC6	2 per week for 2 wks, 1 per week for 6 wks, 1 every second wk for 8 wks	↓muscle sympathetic nerve activity
2010	Johansson (32)	PCOS rats	EA	NR	5 per wk for 4-5 wks	↑GLUT4, ↓high-density lipoprotein/low-density lipoprotein cholesterol
2011	Jedel (33)	PCOS women	EA	CV3, CV6, ST29, SP6, SP9	2 per wk for 2 wks, 1 per wk for 6 wks, 1 every other wk for 8 wks	improve hyperandrogenism and menstrual frequency
2011	Pastore (34)	PCOS women	EA/MA	UB23, UB28, SP6, SP9, PC6, TE5, GV20	2 per wk for 4 wks, 1 per wk for 4 wks	↓ LH/FSH
2012	Billhult (13)	PCOS women	EA/MA	CV3, CV6, ST29, SP6, SP9	2 per wk for 2 wks, 1 per wk for 6 wks, 1 every other week for 8 wks	hope, get result, feel of responsibility, skepticism and proof of effect, feel normal
2012	Feng (35)	PCOS rats	EA/MA	NR	5 per wk for 4–5 wks	restored disturbed oestrous cyclicity
2012	Franasiak (36)	PCOS women	EA/MA	UB23, UB28, SP6, SP9	2 per wk for 4 wks, 1 per wk for 4 wks	not change AMH concentrations
2013	Stener‐Victorin (37)	PCOS women	EA	CV3, CV6, ST9, SP6, and SP9	2 per wk for 2 wks, 1 per wk for 6 wks, 1 every other wk for 8 wks	↓MADRS-S and BSA-S, ↑SF-36, PCOSQ
2013	Johansson (38)	PCOS rats	EA/MA	ST29, SP6	5 per wk for 4-5 wks	↓weight of the subcutaneous fat depot
2013	Rashidi (39)	PCOS women	acupuncture	NR	NR	↑embryo quality
2013	Yu (40)	PCOS women	EA	ST25, CV12, CV6, SP6, BL17, BL32	3 per wk	↓BW, BMI, WHR, FINS, ↑ISI and APN
2013	Johansson (41)	PCOS women	EA/MA	CV3, CV6, ST25, ST29, SP6, SP9, LI4, GV20, LR3, PC6, GV20	2 per wk for 10 –13 wks	↑ovulation frequency
2013	Sun (42)	PCOS rats	EA	CV3, CV4	once daily for 14 consecutive days	↑P450arom, ↓P450c17α
2013	Zheng (43)	PCOS women	acupuncture	NR	once a day for 6 months	↓BMI, WHR, ovarian volume, luteotrophic hormone, LDL-C, T, ↓ LH/FSH, ↑Menstrual frequency and HDL-C
2016	Stener-Victorin (44)	PCOS women	EA	CV4, CV12, ST29, ST34, ST32, SP6, ST36, LI4, CV6, CV10, ST27, SP10, SP6, LR3, PC6	3 per wk over 5 wks	↓HbA1c, ↓circulating and adipose tissue androgens
2016	Ramadoss (45)	PCOS rats	EA	biceps femoris and erector spinae muscle	alternate days for 4-5 wks	↓ sympathetic tone
2017	Benrick (46)	PCOS women	EA	ST27, ST28, ST29, SP6, SP9	NR	↑whole-body glucose uptake, ↑sympathetic/partly
2017	Maliqueo (47)	PCOS rats	EA	NR	5 per wk for 5-6 wks	↑low-density lipoprotein-cholesterol
2018	Kokosar (48)	PCOS women	EA	CV3, CV12, ST29, ST32, ST34, ST36, SP6, LI4	NR	↑sympathetic nervous system ↑whole body glucose uptake
2018	Ma (49)	PCOS rats	EA	ST29, SP6	5 per wk for 4 wks	↑angiogenesis in the antral follicles
2018	Cui (50)	PCOS rats	EA	SP6, ST29, GV20	3 wks	↓global DNA methylation and Dnmt3b expression, ↓LH/FSH, ↑Menstrual frequency and HDL-C
2019	Shi (51)	PCOS rats	EA	CV3, CV4	for 14 consecutive days	↓AMH
2019	Wang (52)	PCOS women	EA	CV3, CV6, ST29, SP6, SP9, LI4, GV20, ST25, LR3, PC6	2 per wk for 16 wks	↑serum NE, ↓5-HT, ↓GABA
2019	Budihastuti (53)	PCOS women	EA	CV3, CV6, ST29, SP6, LI4, ST36	2 per wk for 6 wks	↑oocytes’ growth
2019	Rouhani (54)	PCOS women	EA	ST21, ST25, ST28, ST29, REN12, REN6, REN4, SP9, SP6, ST40	20 times	↓body fat and BMI, WHR, fasting insulin, ↑insulin sensitivity
2020	Benrick (55)	PCOS women	EA	CV3, CV12, ST29, ST 32 ST34, ST36, SP6, LI4	NR	normalize gene expression in skeletal muscle
2020	Peng (56)	PCOS rats	EA	ST29, SP6	5 wks	↓sterol regulatory element binding protein 1
2020	Li (57)	PCOS women	EA/MA	ST29, CV3, CV12, ST34, ST33, SP6, ST36, ST27, CV6, CV10, SP10, SP6, LR3	3 per wk for 6 months	↓homeostatic model assessment for insulin resistance
2020	Peng (12)	PCOS rats	EA	ST29, SP6	5 wks	↑autophagy
2020	Tong (58)	PCOS rats	EA	ST29, SP6	5 per wk for 4 wks	regulating ovarian innervation
2020	Xu (59)	PCOS rats	EA	CV3	14 consecutive days	↓T, FAI, LH, LH/FSH, AMH, INHB, FINS, ↑E2, FSH, and SHBG
2021	Budihastuti (60)	PCOS women	EA	CV3, CV6, ST29, SP6, LI4, ST36	2 per wk for 6 wks	↑folliculogenesis/endometrial thickness
2021	Chen (61)	PCOS rats	acupuncture	CV4, RN3, CV6, SP6, EX-CA1	each day over 11 days	↓LncMEG3, PI3K/AKT/mTOR pathway, granulosa cell autophagy
2021	Dong (62)	PCOS women	EA	CV3, CV6, ST29, SP6, SP9, LI4, GV20, ST25, LR3, PC6	16 wks	↓weight, BMI, hipline, homeostatic model assessment of insulin sensitivity, ↓visfatin, HDL-C, WHR, fasting glucose, ↑resistin and IL-6
2021	Wang (63)	PCOS rats	EA	CV6, SP6, ST36	5 per wk for 2 wks	modulate the kisspeptin system
2021	Xiang (64)	PCOS women	EA	RN12, ST25, SP15, GB26, CV6, CV4, SP10, ST40, ST36, SP9	2 per wk until the day of oocyte collection	↑ IRS-1/PI3K/GLUT4 signaling pathway
2021	Wu (65)	PCOS women	acupuncture	ST36, CV4	2 per wk for 3 months	↓miR-32-3p, ↑PLA2G4A
2021	Zhao (66)	PCOS women	EA	NR	3 per wk for 12 wks	↓LH, AMH, T, ↑P450arom, E2
2022	Dong (67)	PCOS women	EA	CV3, CV6, GV20, ST29, ST25, SP6, SP9, LI4, LR3, PC6	2 per wk with a maximum of 32 treatments	↓DHEA secretion and the acne score
2022	Pan (68)	PCOS women	acupuncture	RN4, EX-CA1, ST29, ST36, SP6, RN6, RN12, DU20, ST25, KI3, KI6, LR3, SP10, PC6	2 per wk for three menstrual cycles	↑pregnancy and ovulation rate
2022	Zhang (69)	PCOS rats	EA	ST29, SP6, LR3, PC6	NR	↓white adipose tissue, ↑brown adipose tissue

AMH, anti-Müllerian hormone; APN, adiponectin; AR, androgen receptor; BMI, body mass index; BSA-S, Brief Scale for Anxiety; BW, body weight; DHEA, Dehydroepiandrosterone; EA, electro‐acupuncture; E2, estradiol; FAI, free androgen index; FINS, fasting serum insulin; FSH, follicle-stimulating hormone; GLUT4, Glucose transporter type 4; GnRH, gonadotropin releasing hormone; IL, Interleukin; INHB, inhibin B; ISN, insulin sensitivity index; LDL-C, LH, luteinizing hormone; MA, manual acupuncture; MADRS-S, Montgomery Åsberg Depression Rating Scale; NGF, nerve growth factor; NR, not reported; P450arom, P-450 aromatase; PCOSQ, PCOS Questionnaire; SHBG, sex hormone-binding globulin; SF-36, Swedish Short-Form 36; T, testosterone; WHR, waist-hip ratio; wk, week; wks, weeks.

## The etiology of PCOS

The exact etiology of PCOS is still unclear because of the complicated pathophysiological processes. Mounting evidence suggests that PCOS may be related to hyperandrogenism, insulin resistance, genetic factors, and negative emotions. Hyperandrogenemia plays a vital role in the pathogenesis of PCOS (70). Androgen stimulation may increase the release of gonadotropin-releasing hormone (GnRH), which may lead to an increase in the frequency and amplitude of luteinizing hormone (LH) pulses. Excessive LH release, in turn, may cause excessive production of androgens (71). Moreover, the low levels of follicle-stimulating hormone (FSH) and inadequate conversion of androgen to estradiol prevent the recruitment of dominant follicles, leading to anovulation (71–73). Insulin resistance is another important factor in the pathogenesis of PCOS (74). An abnormal insulin signaling pathway was found in the ovarian tissues of PCOS patients. This may be because prolonged hyperinsulinemia activates the mTOR/S6 kinase pathway, which enhances the serine phosphorylation of IRS-1 and eventually induces insulin resistance in the hypothalamus (75, 76). In addition, insulin levels are also positively correlated with androgen levels in PCOS patients (77). The secretion of androgens increased in PCOS patients under insulin stimulation, which enhanced the activity of cytochrome P450c17α hydroxylase and then increased androgen production. Adiponectin, an adipocyte-specific protein that regulates insulin sensitivity and glucose catabolism, have been found decreased in patients with PCOS (78, 79). Furthermore, high insulin levels in PCOS patients may also accelerate the pulse of LH secretion and stimulate the synthesis of androgens by follicular membrane cells, resulting in hyperandrogenemia and anovulation (80). The incidence of PCOS is often clustered in families, and first-degree relatives are at higher risk (81). Genes such as the CYP17 gene, androgen receptor gene, and SHBG gene have been confirmed to be involved in androgen metabolism (82–84). Recent studies have shown that insulin receptor genes (IRS1 and IRS2) are associated with the incidence of PCOS (85). Chronic negative emotions such as depression and low self-esteem could make the body in a state of stress. Such emotions can directly inhibit the hypothalamic-pituitary-adrenal axis, leading to obstacles in the HPO axis regulation mechanism and ovarian dysfunction, which induce PCOS (86).

## Acupuncture-A possible treatment for PCOS

**Figure 2:**
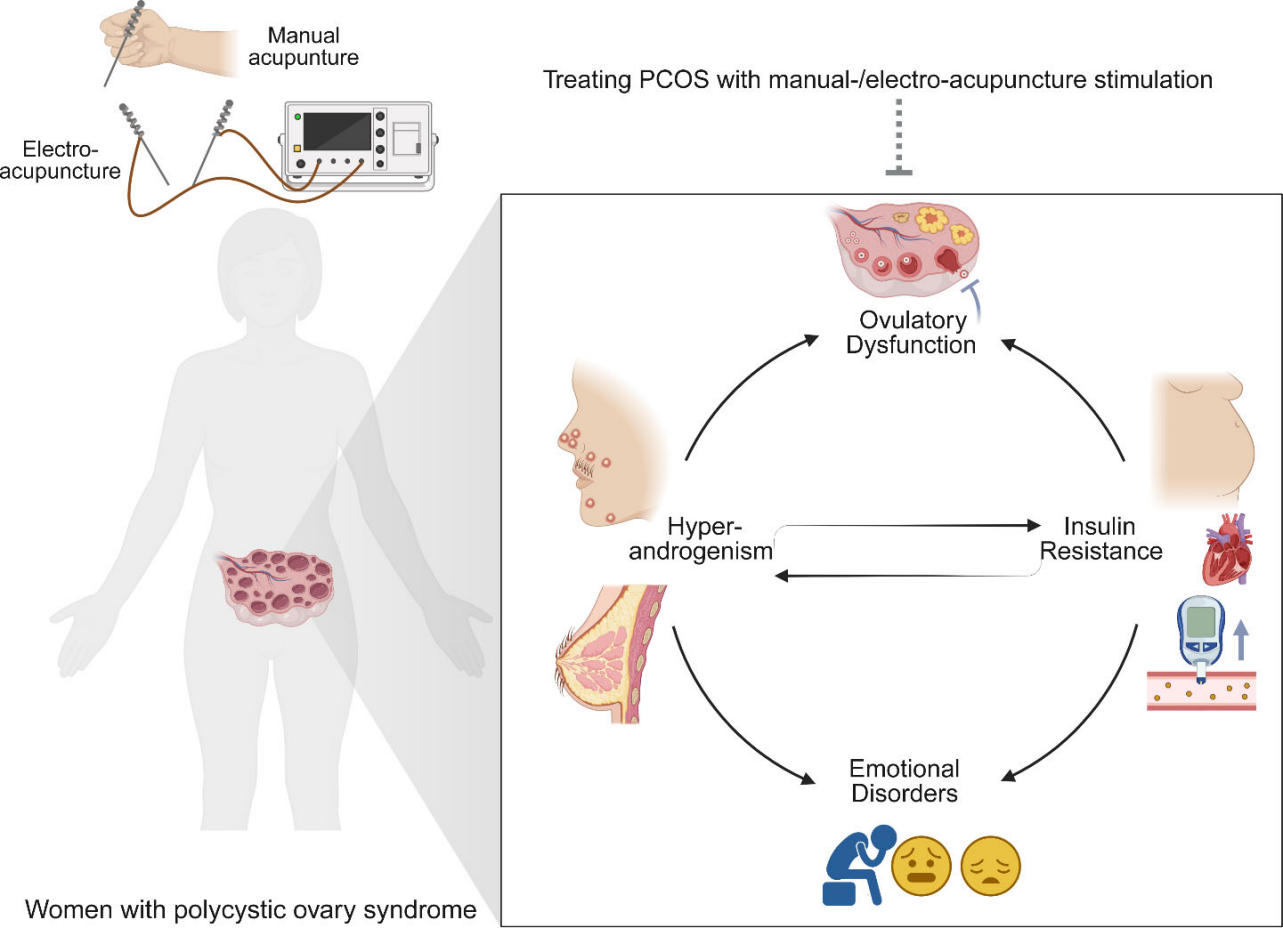
Acupuncture treatment attenuates the major clinical manifestations of polycystic ovary syndrome (PCOS). PCOS is featured by hyperandrogenism (hirsutism, acne, alopecia, etc.), ovulatory dysfunction, polycystic ovaries, obesity, insulin resistance, metabolic dysfunction, and emotional disorders. Manual-/electro-acupuncture improves the PCOS-related symptoms through different biological mechanisms.

Acupuncture, a representative of traditional Chinese medicine, has been widely used for treating diseases in China for at least 2,000 years. Currently, acupuncture is increasingly accepted as a complementary therapy for many disorders worldwide (87). The effectiveness of acupuncture for diseases such as chronic prostatitis, chronic musculoskeletal pain, and chronic severe functional constipation, etc. have been confirmed by many high-quality randomized controlled trials (88–90). Electroacupuncture (EA) is a new form of acupuncture treatment in which acupuncture is combined with electrical stimulation. Multiple clinical trials have shown that manual acupuncture and EA are both effective for treating PCOS (48, 57, 64, 65, 68). The effects of acupuncture for PCOS involved improvement in ovulation rate, pregnancy rate, insulin resistance, negative emotion, sexual hormone disturbance, and lipid metabolism dysfunction (**Figure 2**) (91).

Acupuncture therapy has been used as a complementary and alternative treatment for oligo/anovulatory women with PCOS (91). Studies have revealed that acupuncture might reduce cortisol concentrations and regulate central and peripheral β‐endorphin production and secretion (92). Considering that acupuncture has a potential effect on β-endorphin, which can impact GnRH secretion and levels, it is postulated that acupuncture may play an important role in improving ovulation induction and fertility. In 2016, a systematic review including five randomized controlled trials (RCTs) with 413 women reported insufficient evidence to support the use of acupuncture for the treatment of ovulation disorders in women with PCOS (93). Further in 2019, the updated review added three other new RCTs with a total of 1546 women covering the uncertainty of the effect of acupuncture on the live birth rate, multiple pregnancy rate and ovulation rate compared to sham acupuncture (91). However, acupuncture may ameliorate the restoration of regular menstrual periods. In recent years, accumulating scientific studies have investigated the acupuncture meridians and the neuroendocrinological aspects of the meridians, considering that acupuncture may have a role in normalizing the HPO axis, which in turn influences the menstruation cycle pattern (91, 94). In addition, the evolving omics techniques and emerging analysis tools of biological information may facilitate acupuncture research and help to reveal the mechanisms of acupuncture action on PCOS.

## Evidence-based study of acupuncture for PCOS

A lot of systematic reviews and meta-analyses have been performed to provide evidence-based information in this field. A Cochrane systematic review have been conducted by Lim et al. in 2011, and updated in 2016 and 2019 (91, 93, 95). These studies assessed the effectiveness of acupuncture treatment for oligo/anovulatory women with PCOS for both fertility and symptom control. They concluded that the efficacy of acupuncture on pregnancy outcomes in PCOS patients was uncertain due to the limited number of RCTs and the low quality of evidence (91). A systematic review in 2017 revealed that acupuncture is likely to improve ovulation rate and menstruation rate, but the level of evidence was low (96). In 2020, Wu et al. believed that there was no sufficient evidence supporting the effectiveness of acupuncture to promote live birth, pregnancy, and ovulation in PCOS patients (22). Interestingly, this systematic review suggested that acupuncture could promote the recovery of menstrual cycles as well as downregulate the levels of LH and testosterone in PCOS patients. A recent systematic review showed that acupuncture combined with metformin improved pregnancy rate, ovulation rate, and insulin resistance in PCOS patients compared to using metformin alone (97). Another study found that acupuncture combined with moxibustion improved pregnancy, ovulation, and miscarriage rates, as well as the levels of some sex hormones and metabolic indicators (98). The effectiveness of acupuncture on PCOS patients undergoing *in vitro* fertilization (IVF) or intracytoplasmic sperm injection (ICSI) was also evaluated by a systematic review (99). The results showed that acupuncture may increase the clinical pregnancy rate and ongoing pregnancy rate and decrease the risk of ovarian hyperstimulation syndrome in patients with PCOS undergoing IVF or ICSI. A systematic review by Zheng et al. found that acupuncture was relatively effective in improving glucose metabolism and insulin sensitivity in patients with PCOS (100). Furthermore, a systematic review assessed the efficacy of acupuncture on animal models with PCOS (23). They found that a definite conclusion was difficult to draw because the methodology was weak and heterogeneity was high. The methodological and reporting quality of systematic reviews on acupuncture treatment for patients with PCOS was also evaluated by a systematic review. This study demonstrated poor methodological and reporting quality of systematic reviews assessing acupuncture in patients with PCOS (101). Information of these systematic reviews and meta-analyses is shown in **Table 2**.

**Table 2:** Characteristics of systematic reviews and meta-analyses assessing acupuncture for PCOS.

Year	Author	Country	Subject	Comparison	Indicator
2011	Lim (95)	Australia	RCTs	NA	NA
2016	Lim (93)	Australia	RCTs	acupuncture VS sham acupuncture, electroacupuncture VS physical exercise, electroacupuncture VS no intervention, acupuncture VS relaxation, acupuncture VS clomiphene	live birth rate, ovulation rate, clinical pregnancy rate, restoration of menstruation, multiple pregnancy, miscarriage, and adverse events
2017	Jo (99)	South Korea	RCTs	acupuncture VS sham acupuncture, acupuncture VS no treatment, acupuncture VS other treatments	clinical pregnancy rate, live birth rate, ongoing pregnancy rate, incidence of OHSS, adverse events
2017	Jo (96)	South Korea	RCTs	acupuncture VS sham acupuncture, acupuncture VS medication, acupuncture VS no treatment	ovulation rate, menstruation rate, LH, LH/FSH ratio, testosterone, fasting insulin, and pregnancy rate
2018	Luo (101)	China	systematic reviews	NA	methodological and reporting quality
2019	Lim (91)	Australia	RCTs	acupuncture VS sham acupuncture, acupuncture VS relaxation, acupuncture VS clomiphene; low-frequency electroacupuncture VS physical exercise or no intervention, acupuncture VS Diane-35	live birth rate, multiple pregnancy rate, ovulation rate, clinical pregnancy rate, restored regular menstruation period, miscarriage rate, and adverse events
2020	Wu (22)	China	RCTs	acupuncture VS sham acupuncture, acupuncture VS clomiphene citrate, acupuncture VS letrozole, acupuncture VS metformin, acupuncture VS Daine-35, acupuncture VS Chinese medicine, acupuncture VS treatment	live birth rate, pregnancy, ovulation, recovery of menstrual period and hormone levels
2021	Zheng (100)	China	RCTs	acupuncture VS no acupuncture	body mass index, waist-to-hip ratio, fasting plasma glucose, insulin resistance, triglycerides
2021	Li (23)	China	Animal studies	acupuncture plus PCOS animals VS PCOS animals	insulin resistance, testosterone, LH, LH/FSH ratio, fasting blood sample, fasting insulin, and body weight
2022	Li (98)	China	RCTs	acupuncture combined with moxibustion plus basic treatment VS basic treatment	pregnancy, ovulation, miscarriage, sex hormones, and metabolic disorders
2022	Chen (97)	China	RCTs	acupuncture plus metformin VS metformin	pregnancy rate, ovulation rate, insulin resistance

FSH, follicular stimulating hormone; LH, luteinizing hormone; NA, not available; OHSS, ovarian hyperstimulation syndrome; RCTs, randomized controlled trials; VS, versus.

## Potential mechanisms of acupuncture affecting pcos-related symptoms

### Ovulatory dysfunction

Ovulatory dysfunction is one of the most sovereign characteristics of PCOS (102). Oocyte quality has been proved important for reproductive potential in women with PCOS (103). EA was proved to be effective in improving oocyte quality and embryonic development potential in infertile patients with PCOS (64). Upregulation of the IRS-1/PI3K/GLUT4 signaling pathway appears to be involved in the effect of EA. A similar study demonstrated that EA improved abnormal follicular development in PCOS patients by inhibiting the overexpression of AMH and increasing the expression of P450arom (66). The protective effect of EA on follicle growth in patients with PCOS was further confirmed by another clinical study (53, 60). In addition, acupuncture at an early stage of oocyte recruitment improved embryo quality in PCOS patients undergoing *in vitro* fertilization (39). A recent animal study revealed that acupuncture improved ovulation disorder by downregulating LncMEG3 expression, inhibiting the PI3K/AKT/mTOR pathway, and reducing granulosa cell autophagy (61). EA was also reported to improve follicular arrest in PCOS rats by decreasing the overexpression of AMH to normalize FSH and AMH imbalance in granulosa cells (51). Follicular maturation may be affected by endogenous ovarian angiogenesis, which may be another mechanism underlying EA in the treatment of PCOS (49). Interestingly, another animal study demonstrated that EA upregulates the numbers of preovulatory follicles and corpora lutea by increasing innervation of blood vessels near the hilum (58).

PCOS is a multi-symptom disorder linked with a range of reproductive hormonal disturbances (104). A recent clinical study showed that acupuncture improved the pregnancy rate and ovulation rate in infertile women with PCOS, and the effect may be related to the modulation of acupuncture on sex hormones disturbance (68). In another study, acupuncture induced a higher ovulation frequency in lean/overweight PCOS women (41). Meanwhile, acupuncture also reduced the serum levels of ovarian and adrenal sex steroid. In an animal study, EA improved the disturbed estrous cycles and upregulated the number of corpora lutea and area of the ovary in a pubertal rat model of PCOS (63). The increased LH and decreased estradiol and GnRH were all normalized by EA in this study. Furthermore, EA attenuated the upregulation of kisspeptin protein level in the arcuate nucleus, which might explain the efficacy of EA (63).

### Hyperandrogenism

Evidence suggests that hyperandrogenism is an important clinical feature and mechanism of PCOS (105). Many clinical studies have shown that acupuncture can lower the serum level of testosterone in PCOS women (33, 41, 68, 106). In a study, the circulating and adipose tissue androgen levels in PCOS patients were decreased by EA (44). The effect of EA may be associated with decreased level of hemoglobin A1C. Another study showed that EA improved hyperandrogenism in PCOS patients, and regulation of AMH and P450arom may be involved in the potential mechanism of EA (66). In an animal study, acupuncture inhibited excessive androgen secretion in a rat model of PCOS. The efficacy of acupuncture may be related to the inhibitory effect on overexpression of androgen receptor and connexin 43 (59). Another animal study revealed that EA improved the local ovarian hyperandrogenic environment, probably through increasing P450arom level and decreasing P450C17a level (42). In addition, research showed that EA decreased the overexpression of AMH and regulated FSH and AMH imbalance in granulosa cells, improving hyperandrogenism in a rat model of PCOS (51). Low-frequency EA also decreased serum testosterone in rats with PCOS, and the efficacy may be mediated by central opioid receptors such as *Oprk1* and *Oprm1* in the hypothalamic arcuate nucleus (35).

### Insulin resistance and obesity

**Figure 3:**
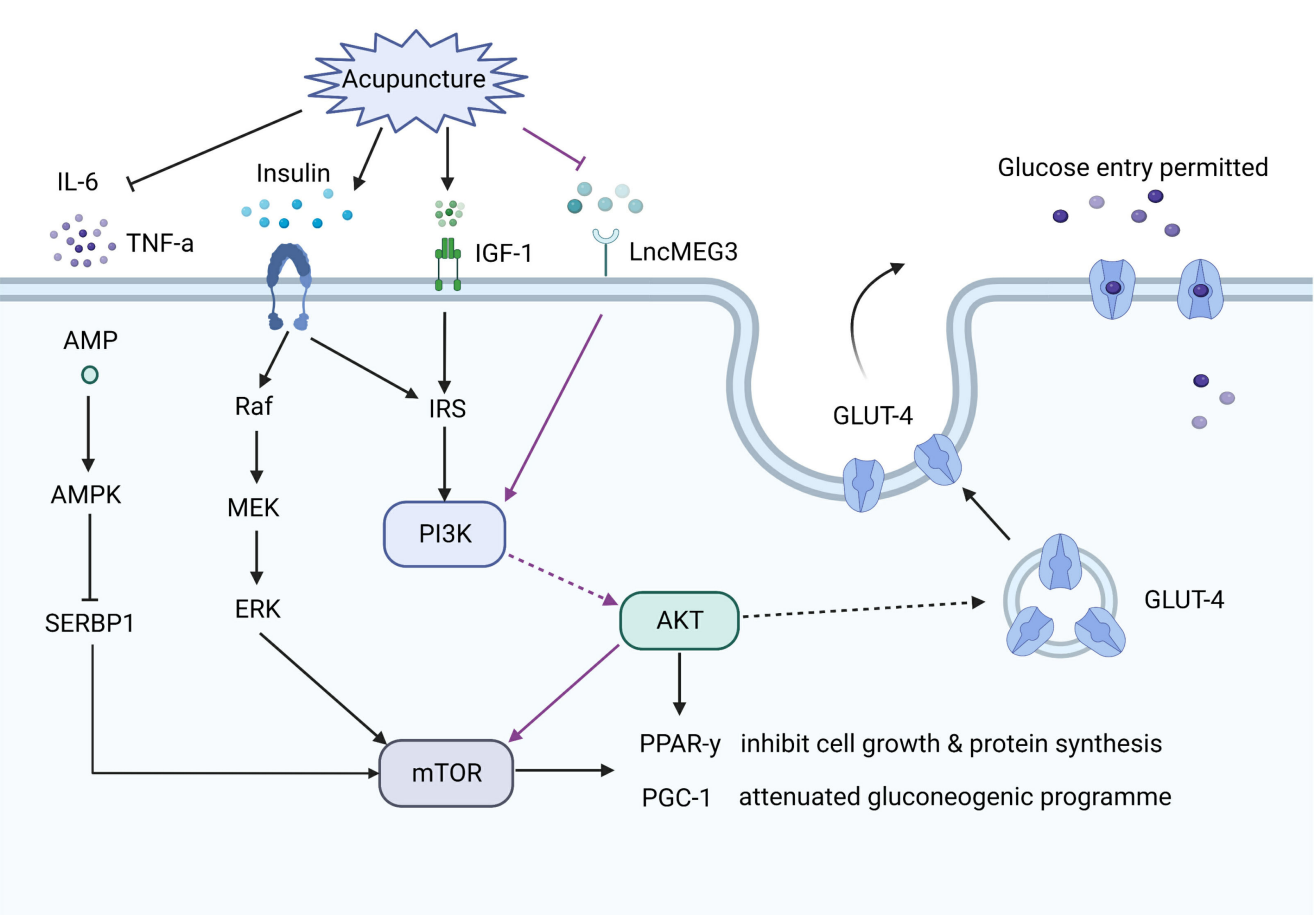
The effect of acupuncture on the insulin pathways. Acupuncture increases glucose transporter 4 (GLUT4) expression *via* upregulating the insulin receptor substrates-1/PI3K/GLUT4 pathway, or inhibiting the PI3K/AKT/mTOR pathway, or activating the adenosine monophosphate activated protein kinase (AMPK) pathway. Acupuncture also decreases the levels of linterleukin-6 and tumor necrosis factor-α, which are involved in insulin resistance.

There is general agreement that PCOS patients are insulin resistant, especially obese PCOS patients (107). Insulin resistance and related hyperinsulinemia may induce both the endocrine and reproductive traits of PCOS (108). The efficiency of acupuncture on insulin resistance in PCOS patients has been confirmed by many clinical studies (43, 57, 64). A recent study demonstrated that EA improved the insulin resistance score compared with the control group in PCOS patients, and the protective effect of EA might be through an upregulation of the IRS-1/PI3K/GLUT4 signaling pathway (64). Abdominal acupuncture also improved insulin resistance in patients with obesity-type PCOS, which may be related to the efficacy of acupuncture treatment on body–mass index, waist-to-hip ratio (WHR), and lipid metabolism dysfunctions (43). Consistent with this study, EA was found to be effective in improving insulin resistance, as well as decreasing WHR and the levels of total cholesterol and low-density lipoprotein (LDL) cholesterol (54, 62). EA was also reported to attenuate insulin resistance by inactivating the mTOR/4E-BP1 signaling pathway in a rat model of PCOS (12). Simultaneously, EA ameliorated mitochondrial dysfunction and endoplasmic reticulum stress by enhancing autophagy. EA improved insulin sensitivity in PCOS models, and this efficacy may be associated with increased plasma insulin-like growth factor-I, increased expression of leptin and interleukin-6 (IL-6) and decreased expression of uncoupling protein 2 in visceral adipose tissue (28). Sterol regulatory element-binding protein-1 (SREBP-1) is an important transcription factor that regulates the expression of genes involved in lipogenesis and glycolysis (109). A study found that EA induced the activation of the AMPK pathway to suppress SREBP-1 expression and finally inhibited insulin resistance, mitochondrial dysfunction and oxidative stress in a PCOS rat model (56). A study investigated whether EA and manual acupuncture have different effects on insulin sensitivity in PCOS rats. They found that EA improved insulin sensitivity in soleus muscle and mesenteric adipose tissue, while manual had a greater effect on glucose tolerance (**Figure 3**) (38).

Glucose and lipid metabolism dysfunctions are found in most obese PCOS patients (110). In a clinical study, acupuncture treatment decreased miR-32-3p levels and increased the expression of PLA2G4A, leading to improvement in PCOS patients with diabetes (65). Gene expression and methylation were analyzed to reveal the mechanism of EA on glucose metabolism dysfunctions in PCOS patients. The results showed that EA regulated gene expression (such as *MSX1* and *SRNX1*) in skeletal muscle in insulin-resistant overweight/obese PCOS women (55). Interestingly, EA increased LDL cholesterol without affecting insulin sensitivity or adipose tissue function in a rat model of PCOS, which might suggest that a balance of sex hormones is necessary to restore metabolic function (47). The same research team also found that EA improved insulin sensitivity and decreased total high-density lipoprotein and LDL cholesterol in the same PCOS models (32). The protein expression of GLUT4 was found to be increased in skeletal muscle, which may be involved in the mechanism of EA on insulin sensitivity. In addition, the gut microbiota is known to be causal in the development of obesity/insulin resistance (111). A recent study showed that EA intervention decreased body weight, probably through regulating gut microbiota in PCOS rats (112). This study also demonstrated that EA can normalize visceral and subcutaneous fat content, brown adipose tissue weight, and glucose tolerance in the PCOS model.

### Emotional disorders

**Figure 4:**
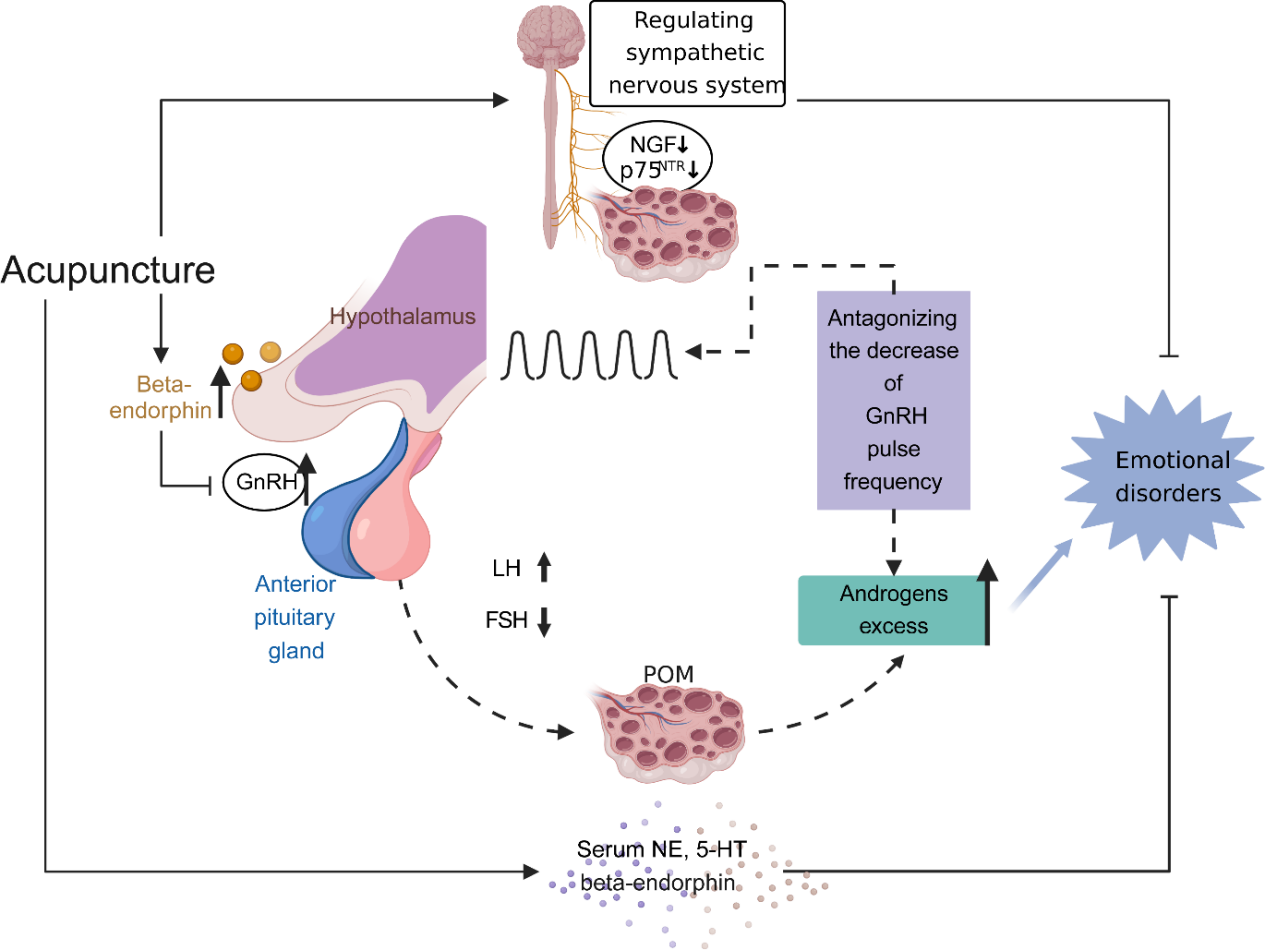
The effect of acupuncture on restoration of the hypothalamic-pituitary-ovarian (HPO) axis and amelioration of emotional disorders. Increased activity and secretion of gonadotropin-releasing hormone (GnRH) with persistently high pulse frequency causes elevated levels of luteinizing hormone (LH) and follicle-stimulating hormone (FSH), contributing to the polycystic ovarian pathology (including impaired follicular development and excess androgen production). Elevated ovarian androgen antagonizes the ability of the progesterone to descend GnRH pulse frequency, leading to a proposed vicious cycle in the HPO axis in polycystic ovary syndrome (PCOS). Meanwhile, androgen excess has an effect on emotion disorders of women with PCOS. Acupuncture modulates central and peripheral β‐endorphin production and secretion, influencing the release of GnRH, then normalising the ratio of LH and FSH, and eventually normalising the HPO axis. Moreover, acupuncture ameliorates emotion disorders of women with PCOS through regulating serum norepinephrine, 5-hydroxytryptamine and β‐endorphin levels, balancing autonomic nervous system, and inhibiting the concentrations of nerve growth factor and the expression of p75 neurotrophin receptor in ovaries.

An increased risk of depression and anxiety has been found in patients with PCOS (113, 114). It has been reported that acupuncture can improve depression and anxiety scores in women with PCOS (37). Interestingly, a recent study revealed that EA appears to improve symptoms of anxiety and depression and regulate the serum levels of norepinephrine (NE) and serotonin (5-HT) in unmarried PCOS patients (52). Anxiety and depression are associated with an autonomic nervous system imbalance (115). Hyperactivation of the sympathetic nervous system is involved in many psychological disorders, such as anxiety and depression (116). Chronic sympathetic overactivity also plays a critical role in the pathogenesis of PCOS (117). A previous clinical study showed that low-frequency EA decreased high muscle sympathetic nerve activity in PCOS patients (31). In addition, another study showed that the efficiency of EA was associated with activation of the sympathetic nervous system (48). In a PCOS rat model, low-frequency EA and physical exercise restored the ovarian expression of markers of sympathetic nervous system activity (30). EA intervention inhibited hyperactivity of the sympathetic nervous system in PCOS rats, which may be related to the inhibitory effects of EA on nerve growth factor (NGF) concentrations in ovaries (24). In addition, the p75 neurotrophin receptor (p75^NTR^) plays a vital role in patterning the sympathetic nervous system during development (118). EA prevented the increase in p75^NTR^ expression, probably by normalizing the sympathetic ovarian response to NGF action (27). Interestingly, the effect of EA on NGF abundance was only found in the ovaries of PCOS rats, but not in the brain (**Figure 4**) (26).

## Acupoint selection

The conception of the acupoint is introduced in Traditional Chinese Medicine (TCM) as the matter that acupuncture acts on the body physiology and relieves symptoms. Increasing evidence has suggested that acupoints are mostly collagen fiber-rich regions, such as intermuscular connective tissue, peri-neurovascular connective tissue, and organ portal and peri-neural connective tissue (26). Moreover, acupoints on different meridians have different effects.

**Figure 5:**
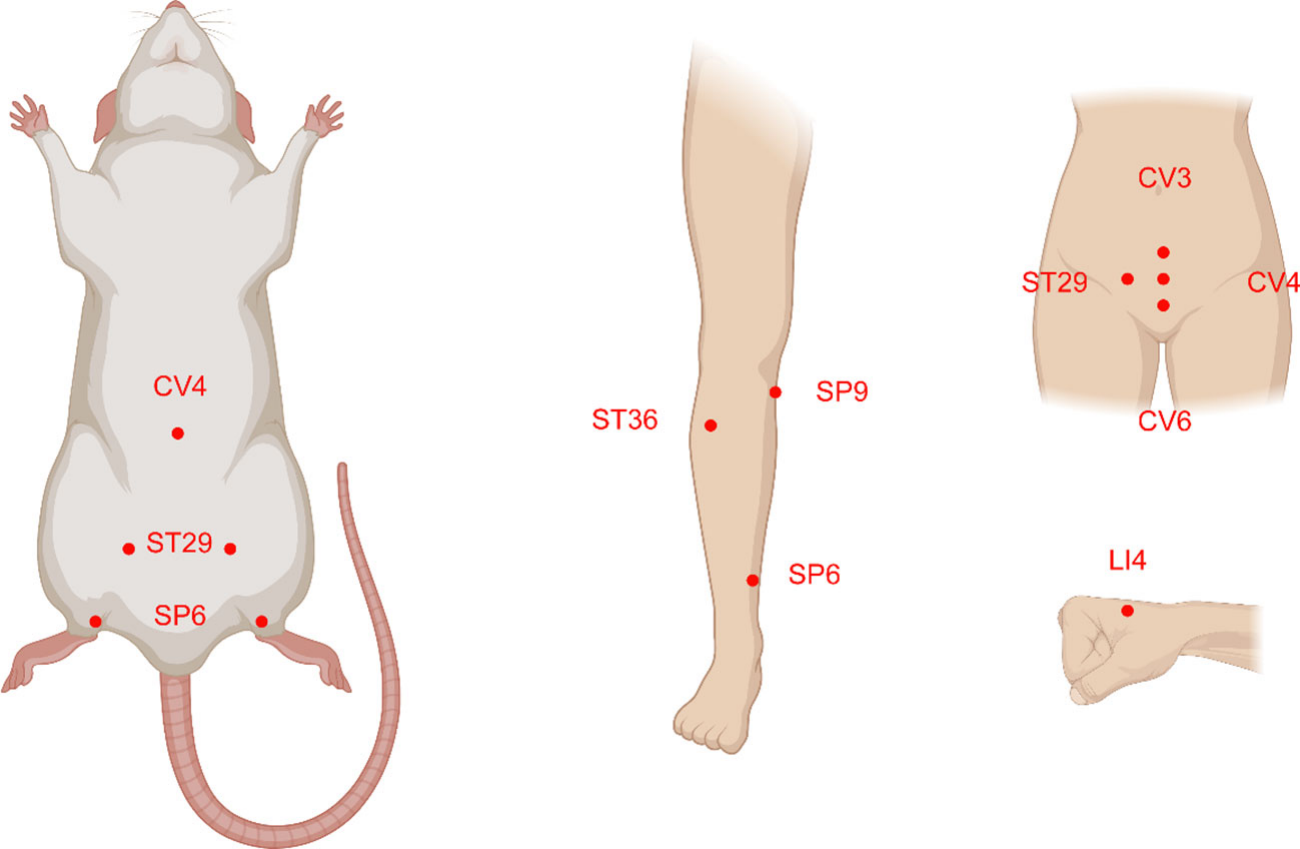
The acupoints common selected and their location distributions in animals and humans with polycystic ovary syndrome (PCOS). The majority of the acupoints are located on the abdomen, upper and lower extremities.

**Figure 6:**
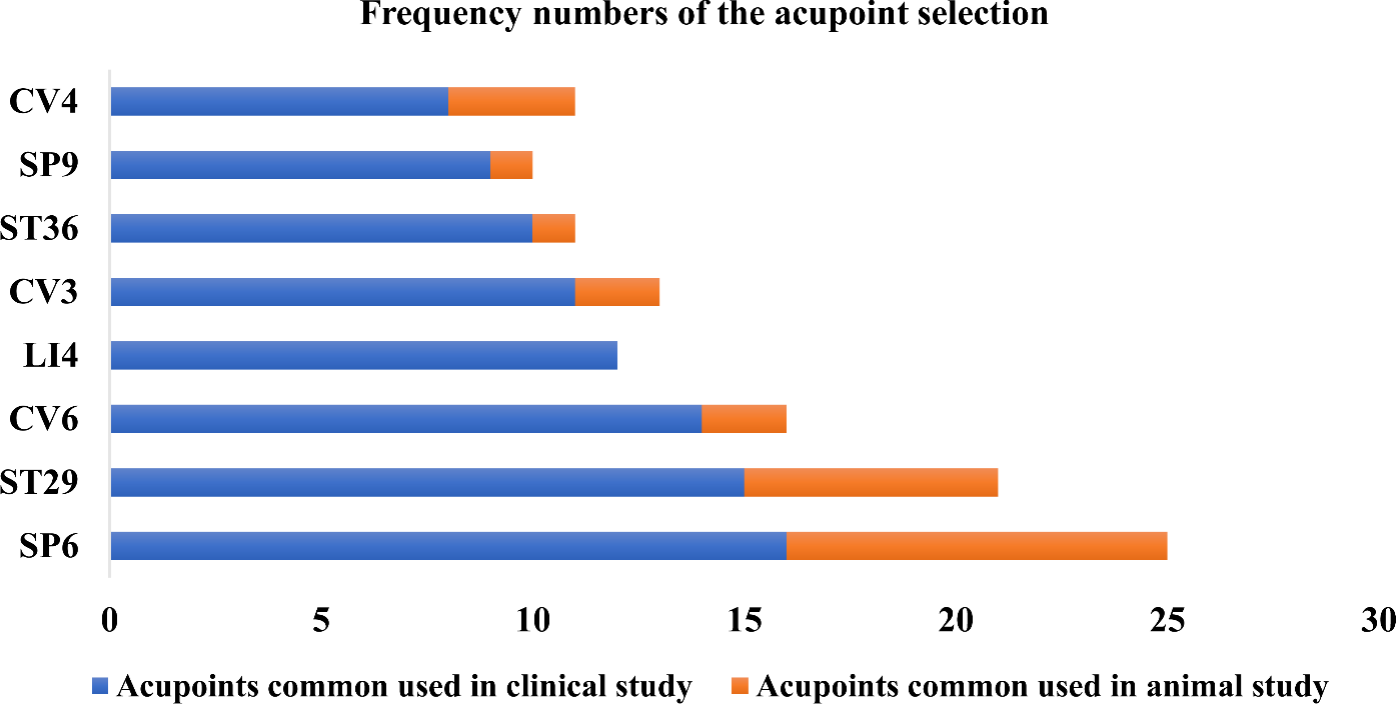
The use frequency of acupoints. SP6 (Sanyinjiao) and ST29 (Guilai) are the most frequently stimulated two acupoints both in animals and humans with polycystic ovary syndrome.

Acupoints SP6, ST29, CV6, LI4, CV3, ST36, SP9, and CV4 have been frequently used in these scientific studies (**Figures 5**, **6**). In clinical applications, the most widely used acupoint was SP6, as it had been selected in sixteen researches. According to the theory of TCM, SP6 (Sanyinjiao) is mainly characterized by the ability to nourish organs, activate blood, soothe the liver and regulate Qi, which can contribute to addressing gynecological problems. The second most involved acupoint was ST29 (Guilai), which was mentioned in fifteen studies. It promotes circulation to remove stasis, regulate menstruation and relieve pain. In addition, CV6, LI4, CV3, ST36, SP9, and CV4 were used in at least eight studies for treating women with PCOS.

Compared with clinical trials, fewer acupoints were stimulated in animal researches. EA was often applied for androgen excess-induced PCOS rat/mouse models. Among the acupoints selected for treatment, SP6 acupoint was the most commonly chosen, either alone or in association with other acupoints, which was consistent with the findings of clinical studies. Additionally, ST29 was more frequently used to mitigate hyperandrogenism and ovulatory dysfunction and to modulate the menstrual cycle. CV4 and ST36 were also frequently used to treat animals with PCOS. Acupoint ST36, in particular, has the ability to tonify Qi and circulation. It was demonstrated that acupuncture at ST36 could lower the levels of IL-6 and tumor necrosis factor (TNF-α) in PCOS animal serum, which might be associated with the ability of acupuncture to inhibit inflammation and oxidative stress (119).

## Discussion

PCOS is a common but heterogeneous disease with symptoms that vary from age to age in patients, typically featuring chronic oligo-anovulation, hyperandrogenism, and/or metabolic disturbance (120). The routine administration after recommending lifestyle modification and some adscititious tips is symptomatic therapy with various agents (121, 122). Patients with PCOS often suffer from a high symptom burden but low tolerance and compliance to pharmacotherapy. Additional regimens need to be explored. With the building availability of acupuncture all over the world, patients with PCOS are increasingly seeking and accepting acupuncture to maintain reproductive health (91). Our previous research manifested that acupuncture can effectively relieve anxiety and depression in patients with PCOS, and its mechanism may be related to the regulation of the levels of serum β-endorphin and androgen (123). In addition, our multinational study protocol on acupuncture or metformin to improve insulin resistance in women with PCOS has been published (124). In recent years, there have been an increasing number of systematic reviews and/or meta-analyses on the effect of acupuncture on PCOS in both patients and animal models (22, 23, 96, 100). This study reviewed the feasibility and efficacy of acupuncture for managing PCOS and summarized the potential mechanisms of acupuncture on treating PCOS. To the best of our knowledge, this is the first detailed review to address acupuncture-specific action on PCOS-related symptoms, including ovulatory dysfunction, hyperandrogenism, insulin resistance, obesity, and emotional disorders.

The disordered hypothalamic–pituitary–ovarian/adrenal axis is one of the most important pathological and physiological states of PCOS (125). As the main metabolic feature of PCOS, insulin resistance is considered to be a crucial pathophysiological basis for the pathogenesis of PCOS (107). Inhibiting phosphoinositide-3 kinase and phosphorylation of IRS-1 impairs insulin signaling by affecting GLUT-4 expression and glucose uptake (126, 127). Another vital pro-inflammatory agent relevant to the pathogenesis of PCOS is adipose tissue (128). It has been proven that adipose tissue-resident macrophages lead to the release of TNF-α and IL-6, which are implicated in the induction of insulin resistance (129). Hyperandrogenism causes the aberration of adipose tissue functions in PCOS. Insulin resistance, hyperandrogenism, chronic low-grade inflammation, and adipose tissue hypertrophy and dysfunction may affect a vicious cycle in the pathophysiology of PCOS (130, 131). Evidence has shown that acupuncture elevates the level of β-endorphin not only in the central endocrine system but also in the peripheral circulation (94), which is associated with both direct and indirect tonic inhibitions of GnRH and subsequent LH release (132). Aberrant sympathetic neurogenic regulation of the ovary is involved in the pathogenesis of PCOS (23), and acupuncture can also inhibit the overexpression of NGF to decrease sympathetic activity, resulting in a restoration to the normal level of the ovarian steroid response to gonadotropins (25). Moreover, acupuncture regulates the phosphorylation of insulin substrates and receptors and inhibits the abnormal expression of signaling pathways, thereby improving metabolic dysfunction such as insulin resistance (32, 56, 133). Acupuncture may also ameliorate cholesterol metabolism, affecting lipid metabolism enzyme activity, inhibiting the synthesis of fatty acids, and then promoting fat decomposition and energy metabolism (100, 134).

Many review articles concerning PCOS and acupuncture have been published during the last 10 years (23, 132, 135–137). However, our present paper is different from those published papers. Firstly, this review summarized current available information from both clinical studies and animal studies. Secondly, the mechanisms of acupuncture on PCOS-related main symptoms (ovulatory dysfunction, hyperandrogenism, insulin resistance, obesity, and negative emotion) were all overviewed. Thirdly, the acupoints that commonly used in PCOS patients and animals were also overviewed in this study. To our knowledge, this is the most comprehensive review that summarized current progress on acupuncture treatment for PCOS.

Some points should be noted. At present, most studies on the effect of acupuncture on PCOS are statistical comparisons, with insufficient depth and breadth of its mechanism of action. System biology and omics techniques have become a new trend, and transcriptomics technology will better analyze the specific expression factors and biological mechanisms of acupuncture treatment. Additionally, there is considerable heterogeneity in terms of animal models (dihydrotestosterone, dehydroepiandrosterone, and testosterone propionate), research intervention (acupoint selection, frequency, electrical current range, pulse width and length of stimulation) and major endpoints (live birth, multiple pregnancy rate, ovulation rate, clinical pregnancy rate, and miscarriage rate), lessening the generalizability of the results from those studies. Moreover, the majority of these studies were conducted in various phenotypes of patients and animal models with PCOS. Although the pathophysiology of the symptoms is similar between several phenotypes and models, there could be differences, making data from one not entirely applicable to the other. As a recent review reported, clinical practice and health policy underuse beneficial acupuncture therapies.

## Comments and future perspectives

At present, a large number of clinical studies have confirmed that acupuncture could improve many symptoms in patients with PCOS. These symptoms include chronic and continuous anovulation, hyperandrogenemia, insulin resistance, negative emotion, glucose and lipid metabolism dysfunction, etc. The effect of acupuncture may be induced by stimulating muscle conduction and chemical signals to induce the central release of key factors through sympathetic nerve conduction and then regulating the female reproductive axis. The selection of acupoints and EA frequency may also impact the therapeutic effect, which needs to be verified by more studies.

With recent advances in technology, the effect of acupuncture could be observed from a more microscopic point of view. Whether the clinical effect of acupuncture is associated with the traditional Chinese meridian theory is still unclear. This needs to be verified and discussed in the next few decades. Clarifying the mechanism of acupuncture in the treatment of PCOS will help to make acupuncture therapy accepted by more people.

## Author contributions

YY and C-CZ contributed to writing the original draft, literature search, and data collection; H-QH contributed to figure presentation and manuscript editing; IF contributed to writing, corrections, and editing; H-LZ contributed to conceiving, designing, editing, and supervising. All authors contributed to this article and approved this submitted version.

## Funding

The work was supported by the Special Grant for Capital Health Research and Development (Grant No. 2022-2-4098), National Natural Science Foundation of China (Grant No. 82174151), Peking University Third Hospital “Key Young Talents” Training Program (Grant No. BYSYFY2021032). The funders have had no role in study design, and will not have any role in data collection and analysis, decision to publish, or preparation of the manuscripts.

## Acknowledgments

We sincerely thank everyone in the department of traditional Chinese medicine, Peking University Third Hospital for discussion and constructive criticism. we would like to thank the BioRender for figure making.

## Conflict of interest

The authors declare that the research was conducted in the absence of any commercial or financial relationships that could be construed as a potential conflict of interest.

## Publisher’s note

All claims expressed in this article are solely those of the authors and do not necessarily represent those of their affiliated organizations, or those of the publisher, the editors and the reviewers. Any product that may be evaluated in this article, or claim that may be made by its manufacturer, is not guaranteed or endorsed by the publisher.
